# Do all patients in the phase I oncology trials need to be hospitalized? Domestic but outstanding issues for globalization of drug development in Japan

**DOI:** 10.1007/s10147-017-1108-z

**Published:** 2017-03-14

**Authors:** Akihiko Shimomura, Shunsuke Kondo, Noriko Kobayashi, Satoru Iwasa, Shigehisa Kitano, Kenji Tamura, Yutaka Fujiwara, Noboru Yamamoto

**Affiliations:** 1 Department of Experimental Therapeutics, National Cancer Center Hospital, 5-1-1, Tsukiji, Chuo-ku, Tokyo, 104-0045 Japan; 2 Department of Breast and Medical Oncology, National Cancer Center Hospital, Tokyo, Japan; 3 Clinical Trial Support Office, National Cancer Center Hospital, Tokyo, Japan

**Keywords:** Phase I trial, Domestic guideline, Severe toxicity, Toxicity management

## Abstract

**Introduction:**

Most trials investigating new drugs around the world, including phase I trials, are conducted in outpatient clinics. However, in Japan, regulatory authority requirements and traditional domestic guidelines often require hospitalization of phase I study participants.

**Patients and methods:**

Patients participating in single-agent phase I clinical trials at National Cancer Center Hospital between December 1996 and August 2014 were monitored. Toxicity requiring hospitalization is defined as toxicity that needs intensive treatment. Study designs were classified into three types: first-in-human (FIH) study, dose-escalation study (conventional dose-escalation study to determine maximum tolerated dose (MTD) in Japanese patients), and dose-finding study (to assess safety and pharmacokinetic profiles up to the MTD previously determined in the West).

**Results:**

A total of 945 patients who participated in a variety of single-agent phase I clinical trials between December 1996 and August 2014 were included in this study. Patients participated in one of three study types: dose-escalation (*n* = 582, 62%), first-in-human (*n* = 129, 14%), or dose-finding (*n* = 234, 25%). A total of 76 study drugs were evaluated as part of this pool of phase I studies. Subdivided by mechanism of action, 20 (26%) were cytotoxic, 50 (66%) were molecularly targeted, and 6 (8%) were immune checkpoint inhibitor. Thirty-six patients (3.8%) had severe toxicities requiring hospitalization during the first cycle. The overall number of toxicities requiring hospitalization and/or grade 4 toxicities during any cycle was 5.0%.

**Conclusions:**

The frequency of severe toxicity that needs to be hospitalized was unexpectedly low. The data did not demonstrate the need for hospitalization in the phase I trials, suggesting that phase I trials in Japan could be conducted in outpatient settings.

## Introduction

Phase I clinical trials of oncologic drugs are conducted to determine the proper dosage of both single-agent and combination therapies. If the therapeutic index is very narrow, it is critical to evaluate the study drug toxicity in dose-escalation studies. Dose-limiting toxicity (DLT) is evaluated during the first cycle of each new dosage level for every trial participant to determine the maximum tolerated dose (MTD). Close monitoring for symptoms as well as physiological and laboratory data are required to determine if DLT is observed.

Clinical trials in Japan and Western countries are carried out under the International Conference on Harmonization of Technical Requirements for Registration of Pharmaceuticals for Human Use (ICH) guidelines [1]. These guidelines do not require hospitalization as part of phase I clinical trials. However, according to Japanese guidelines, participants in phase I oncology clinical trials are required to be hospitalized during the first cycle of treatment to observe toxicity closely for safety reasons [2].

Because the assessment period for DLT is approximately 1 month in most phase I oncology clinical trials, in Japan study participants are required to be hospitalized for 1 month. Phase I clinical oncology trial participants are generally refractory to standard treatments and usually have very limited options. Even though these patients have a good performance status and generally good health, their life expectancy is limited, and consequently, the phase I hospitalization requirement is sometimes in conflict with ongoing end-of-life care. Additionally, the patient is usually charged with the cost of hospitalization, making a patient’s participation in the phase I trial burdensome with respect to time and cost. For all these reasons, it is very difficult to recruit patients to participate in phase I clinical trials. Furthermore, Japan’s hospitalization practice makes it an outlier from standard ICH guidelines, limiting the globalization of Japanese oncology drug development.

Toxicity assessment is an essential objective of a phase I trial. Hyman et al. have developed a nomogram-based model to predict the risk of a patient developing a cycle-one serious drug-related toxicity (SDRT), but its practical use remains very limited [3]. Additionally, the type of investigational drugs upon which this model was largely derived has changed from cytotoxic drugs to molecularly targeted drugs and immune checkpoint inhibitors during the past several decades. Consequently, as the mechanism of action of new cancer drugs has changed, the toxicity profiles and timing of toxicity events have also changed. For example, Postel-Vinay et al. reported that in phase I oncology trials of molecularly targeted drugs, treatment interruption or discontinuation occurs more frequently in the second cycle rather than the first cycle [4].

If the frequency of severe toxicity during first cycle in phase I trials is low, it may be more practical to conduct trials in an outpatient setting or with only minimal hospitalization. Furthermore, minimizing or avoiding hospitalization altogether may facilitate recruitment of participants and accelerate clinical drug development. However, to date, there has been no evaluation of the incidence and risk of severe toxicity in hospitalized patients participating in phase I clinical oncology trials in Japan.

In this study, we surveyed severe toxicity observed in the phase I single-agent clinical trials conducted in our institution and investigated the frequency of toxicity that did or did not require hospitalization.

## Patients and methods

### Patients

Patients who participated in single-agent phase I clinical trials at National Cancer Center Hospital between December 1996 and August 2014 were included in this study. Drug type, treatment course, and toxicity were retrieved from medical records and databases. Severity of toxicity was assessed by Common Terminology Criteria for Adverse Events v. 4.0 [5]. Severe toxicity was classified as requiring hospitalization. Such toxicity requiring hospitalization is defined as follows: toxicity that requires intensive treatment (e.g., continuous oxygen administration, invasive procedure) and/or toxicity that requires intravenous intervention (e.g., fluid therapy, intravenous antibiotics, blood transfusion). Study designs were classified into three types: (1) dose-escalation (conventional dose-escalation study to determine MTD in Japanese patients; FIH study was excluded); (2) first-in-human (FIH); and (3) dose-finding (to assess drug safety and pharmacokinetic profiles up to the MTD previously determined in Western studies).

### Ethical considerations

The present study involving human subjects was approved by the National Cancer Center Institutional Review Board (2014-148). A copy of the letter from the Institutional Review Board is available for review by the Editor of this journal.

## Results

A total of 945 patients participated in the phase I trial from December 1996 to August 2014 at the National Cancer Center Hospital (Tokyo, Japan). Median patient age was 58 years (range, 18–76 years); 537 patients (57%) were men and 408 (43%) were women. Patients were assigned to receive cytotoxic drugs (*n* = 207, 22%), molecularly targeted drugs (*n* = 690, 73%), or immune checkpoint inhibitors (*n* = 48, 5%). Patients participated in one of three study types: dose-escalation (*n* = 582, 61%), first-in-human (*n* = 129, 14%), or dose-finding (*n* = 234, 25%). Tumor types were non-small cell lung cancer (*n* = 248, 28%), colorectal cancer (*n* = 175, 19%), sarcoma (*n* = 115, 12%), esophageal cancer (*n* = 49, 5%), pancreatic cancer (*n* = 45, 5%), bile duct cancer (*n* = 36, 4%), breast cancer (*n* = 35, 4%), and gastric cancer (*n* = 26, 3%) (Table 1).

**Table 1:** Patient characteristics

Patients, *n*	945
Age, years	58 (median), 18–76 (range)
Sex, *n* (%)	
Male	537 (57)
Female	408 (43)
Drug type, *n* (%)	
Cytotoxic	207 (22)
Molecularly targeted	690 (73)
Checkpoint inhibitor	48 (5)
Study type, *n* (%)	
Dose escalation	582 (61)
First in human (FIH)	129 (14)
Dose finding	234 (25)
Disease, *n* (%)	
NSCLC	248 (26)
Colorectal	175 (19)
Sarcoma	115 (12)
Esophagus	49 (5)
Pancreas	45 (5)
Biliary	36 (4)
Breast	35 (4)
Gastric	26 (3)
Melanoma	23 (2)
Ovarian	23 (2)
SCLC	20 (2)
HN	18 (2)
Prostate	18 (2)
Uterine	17 (2)
CUP	10 (1)
Other	87 (9)

*NSCLC* non-small cell lung cancer, *SCLC* small cell lung cancer, *HN* head and neck, *CUP* carcinoma of unknown primary

A total of 76 study drugs were evaluated as part of this pool of phase I studies. Subdivided by mechanism of action, 20 (26%) were cytotoxic, 50 (66%) were molecularly targeted, and 6 (8%) were immune checkpoint inhibitor (Table 2).

**Table 2:** Characteristics of study drugs and clinical studies

Study drug, *n*	76
Study drug, *n* (%)	
Cytotoxic	20 (26)
Molecularly targeted	50 (66)
Checkpoint inhibitor	6 (8)
Study type, *n* (%)	
Dose escalation	44 (57)
First in human	8 (11)
Dose findings	24 (32)

**Fig. 1:**
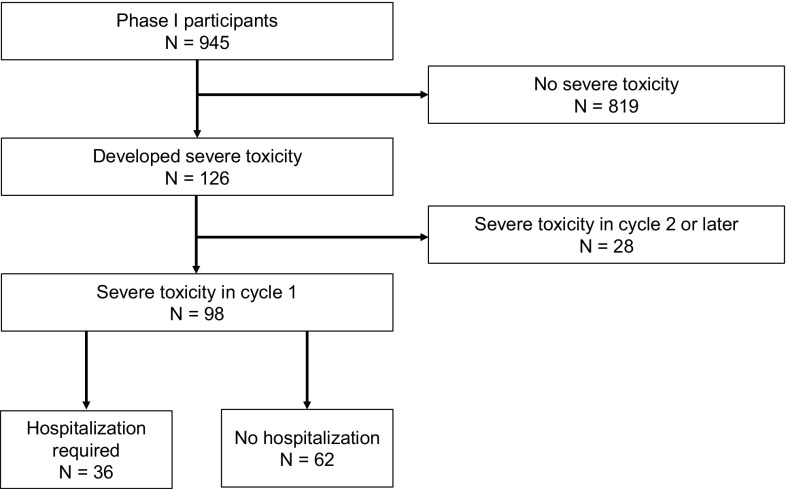
Patient outcomes

Ninety-eight patients (10.4%) developed severe toxicities during the first cycle; a total of 126 patients (13.3%) developed severe toxicities during any cycle. Thirty-six patients (3.8%) had severe toxicities requiring hospitalization during the first cycle (Fig. 1). The overall number of toxicities requiring hospitalization and/or grade 4 toxicities during any cycle was 5.0%. In the first cycle, 31 patients (15.0%) who received cytotoxic drugs and 67 patients (9.7%) who received molecularly targeted drugs developed severe toxicity. However, patients who received immune checkpoint inhibitors did not develop any severe toxicity. Fourteen patients (6.8%) receiving cytotoxic drugs and 22 patients (3.2%) receiving molecularly targeted drugs needed to be hospitalized because of toxicity. For patients receiving cytotoxic agents, 4 hematological (1.9%) and 10 nonhematological (4.8%) toxicity events required hospitalization (Table 3). For patients receiving molecularly targeted drugs, 3 hematological (0.4%) and 19 nonhematological (2.8%) toxicity events requiring hospitalization (Table 3).

**Table 3:** Toxicity by drug type

	Drug type				
	Cytotoxic, *n* = 207		Molecularly targeted, *n* = 690		Checkpoint inhibitor, *n* = 48
Number of severe toxicities in cycle 1, *n* (%)	31 (15)		67 (9.7)		0 (0)
Hospitalization required, *n* (%)					
Hematological	14 (6.8)	4 (1.9)	22 (3.2)	3 (0.4)	0 (0)
Nonhematological		10 (4.8)		19 (2.8)	
No hospitalization required, *n* (%)					
Hematological	17 (8.2)	8 (3.9)	45 (6.5)	6 (0.9)	0 (0)
Nonhematological		9 (4.3)		39 (5.7)

Seventy-three patients (12.5%) in dose-escalation studies, 14 patients (10.9%) in FIH studies, and 11 patients (4.7%) in dose-finding studies developed severe toxicity, including DLT. Twenty-seven (4.6%) dose-escalation study participants, 4 (3.1%) FIH study participants, and 5 (2.1%) dose-finding study participants needed hospitalization for toxicity (Table 4).

**Table 4:** Toxicity by study type

	Dose escalation, *n* = 582	FIH, *n* = 129	Dose finding, *n* = 234
Number of severe toxicities in cycle 1, *n* (%)	73 (12.5)	14 (10.9)	11 (4.7)
Required hospitalization, *n* (%)	27 (4.6)	4 (3.1)	5 (2.1)
Required hospitalization and/or G4, *n* (%)	34 (5.8)	7 (5.4)	6 (2.6)

*FIH* first-in-human

The observed toxicity profile included increase in aminotransferase (*n* = 39, 17.3%), neutropenia (*n* = 22, 9.7%), thrombocytopenia (*n* = 20, 8.8%), anorexia (*n* = 13, 5.8%), and proteinuria (*n* = 13, 5.8%). During the first cycle, toxicities requiring hospitalization included thrombocytopenia (*n* = 11, 19.0%), febrile neutropenia (*n* = 9, 15.5%), ileus/bowel obstruction (*n* = 5, 8.6%), arrhythmia (*n* = 3, 5.2%), and pneumonia (*n* = 3, 5.2%) (Table 5).

**Table 5:** Adverse events (AEs) observed during trial

	Any AEs		AEs requiring hospitalization in cycle 1	
	*n*	%	*n*	%
Aminotransferase increased	39	17.3	4	6.9
Neutropenia	22	9.7	0	0.0
Thrombocytopenia	20	8.8	11	19.0
Anorexia	13	5.8	1	1.7
Proteinuria	13	5.8	0	0.0
Nausea	10	4.4	0	0.0
Febrile neutropenia	9	4.0	9	15.5
Anemia	7	3.1	2	3.4
Malaise	7	3.1	1	1.7
CPK increased	5	2.2	0	0.0
Diarrhea	5	2.2	0	0.0
Ileus	5	2.2	5	8.6
Rash	5	2.2	1	1.7
T-Bil increased	5	2.2	0	0.0
WBC decreased	5	2.2	0	0.0
Hypertension	4	1.8	0	0.0
GGT increased	4	1.8	0	0.0
Mucositis	4	1.8	1	1.7
Vomiting	4	1.8	2	3.4
Arrhythmia	3	1.4	3	5.2
Pneumonitis	3	1.4	3	5.2
Colitis	2	0.9	2	3.4
Hypoxia	2	0.9	2	3.4
Infection	2	0.9	2	3.4
CIPN	2	0.9	0	0.0
QTc prolonged	2	0.9	0	0.0
Unacceptable by patients	2	0.9	0	0.0
AMY increased	1	0.4	0	0.0
Constipation	1	0.4	1	1.7
Creatinine increased	1	0.4	0	0.0
Dehydration	1	0.4	1	1.7
Drug fever	1	0.4	0	0.0
Dry skin	1	0.4	0	0.0
DVT	1	0.4	0	0.0
Dyspnea	1	0.4	1	1.7
ECG abnormality	1	0.4	0	0.0
Edema	1	0.4	0	0.0
Hearing disorder	1	0.4	0	0.0
Hyperglycemia	1	0.4	1	1.7
Hypoalbuminemia	1	0.4	0	0.0
Hypotension	1	0.4	1	1.7
Lipase increased	1	0.4	0	0.0
Muscle weakness, extremity lower	1	0.4	0	0.0
Perforation	1	0.4	1	1.7
Pericardial effusion	1	0.4	1	1.7
Pleural effusion	1	0.4	1	1.7
Pneumonia	1	0.4	1	1.7
Pyrexia	1	0.4	0	0.0
Uric acid increased	1	0.4	0	0.0
Total	226	100	58	100

*AEs* adverse events, *CPK* creatinine phosphokinase, *T-Bil* total bilirubin, *WBC* white blood cell, *GGT* gamma-glutamyltransferase, *CIPN* chemotherapy-induced peripheral neuropathy, *AMY* amylase, *ECG* electric cardiogram, *DVT* deep vein thrombosis

## Discussion

Toxicity evaluation is one of the primary objectives of phase I oncology trials because such studies help define the recommended dose for further studies. The type and timing of toxicity can differ depending on a variety of factors including different dose levels, the class and mechanism of action of the study drug, and the route of administration [6, 7]. As new cancer targets have emerged over the past several decades, there is now a much wider array of investigational drugs with new mechanisms of action. As such, classical a “3 + 3 design” may be not sufficient to evaluate toxicities in the current era [7–9].

A modeling approach using toxicity data from 3104 participants in 127 phase I trials between 2000 and 2010 to estimate the risk of SDRT has been reported [3]. In this study, SDRT was defined as grade ≥4 hematological toxicity or grade ≥3 nonhematological toxicity attributed, at least possibly, to study drug(s). The parameters used in this empirical model to predict SDRT risk included performance status, white blood cell count, creatinine clearance, serum albumin, alanine aminotransferase, aspartate aminotransferase, alkaline phosphatase, number of study drug(s), class of study drug, dose level, and constitutional symptoms. Although the resulting nomogram can be useful in predicting a patient’s risk for SDRT at the time of enrollment, its usage remains limited. In our study, we assessed every toxicity observed after study enrollment regardless of study drug causality. Frequent severe toxicities were increased aminotransferase, neutropenia, and thrombocytopenia. Toxicities requiring hospitalization were hematological, such as thrombocytopenia and febrile neutropenia, a tendency similar to that previously observed [3]. Toxicity requiring hospitalization was more frequent in studies involving cytotoxic drugs compared to those using molecularly targeted drugs (15.9% vs. 9.4%, respectively) (Table 3). This result is consistent with other reported observations that molecularly targeted drugs are negatively correlated with SDRT. In this study, we did not observe severe toxicity in patients who received immune checkpoint drugs. However, there are some reports of severe toxicity associated with this class of drugs [10], and the lack of observed severe toxicity in our study may be because only a small number of patients received immune checkpoint drugs compared to the other drug classes.

Severe toxicity was observed in 10.4% of all participants during the first cycle and in 3.2% of all participants during the second or later cycles, a result similar to a previous report. Thus, it is important to observe toxicity carefully not only during the first cycle but in later cycles as well. We observed that 3.8% of participants experienced severe toxicity requiring hospitalization, and that 5.0% of all participants experienced either grade 4 toxicity or severe toxicity requiring hospitalization. This frequency was unexpectedly low and was independent of drug class. This overall low severe toxicity rate suggests that toxicity can be safely monitored in an outpatient setting and that hospitalization during the entire clinical study period is not necessary in most studies. Nonetheless, toxicity observation is still important after the DLT evaluation period. Moreover, it is important to continue careful monitoring for toxicity throughout the course of a phase I trial of immune checkpoint inhibitors because such drugs may have delayed adverse effects [10].

The frequency of toxicity by study types was similar in both the dose-escalation and FIH studies (12.4% and 10.4%, respectively) (Table 4), possibly because FIH studies start at much lower dosage levels (e.g., 0.1 LD_10_ of mice) compared to the doses used in dose-escalation studies. Severe toxicity in dose-finding studies was low (4.7%), likely because the dose level in these studied may not reach the MTD. Consequently, in phase I oncology dose-escalation and dose-finding studies, hospitalization during the first cycle should not be mandatory. In contrast, in FIH studies conducted to determine MTD, careful observation is required because the drug’s toxicity profile is not well characterized, and hospitalization is one option to ensure patient safety. In FIH studies in Japan for drugs studied in earlier Western trials, it can be useful to use the Western clinical data to design phase I trials that reduce the risk of severe toxicity because it has been observed that the both the MTD and DLT frequency tends to be very similar between Western and Japanese patients [11].

The present study has some limitations. It is a retrospective study, and there is no analysis by design (e.g., conventional “3 + 3” design, accelerated titration design, etc.) or by dosage levels. Of particular note, there is no standardization regarding the type of toxicity requiring hospitalization in Japan compared to other countries. We employed the definition described in the Methods, although it should be noted that hematological toxicity can be managed in an outpatient setting by blood transfusion with close monitoring in some cases. An effort between clinical investigators, regulatory authority, and other key stakeholders to identify a consensus definition of “toxicity requiring hospitalization” is warranted.

## Conclusion

Our results indicate that there is a low rate of severe toxicity requiring hospitalization in phase I oncology trials in Japan. Consequently, the conventional approach requiring participants to be hospitalized during the first cycle of the phase I trial is not necessary. Rather, the outpatient setting should be considered for phase I clinical trials in Japan.
